# Astaxanthin as a Peroxisome Proliferator-Activated Receptor (PPAR) Modulator: Its Therapeutic Implications

**DOI:** 10.3390/md17040242

**Published:** 2019-04-23

**Authors:** Chang-Ik Choi

**Affiliations:** College of Pharmacy, Dongguk University-Seoul, Goyang 10326, Korea; cichoi@dongguk.edu; Tel.: +82-31-961-5230

**Keywords:** astaxanthin, peroxisome proliferator-activated receptors (PPARs), anti-inflammation, anticancer, lipid and glucose metabolism, PPAR modulator

## Abstract

Peroxisome proliferator-activated receptors (PPARs) are part of the nuclear hormone receptors superfamily that plays a pivotal role in functions such as glucose and lipid homeostasis. Astaxanthin (ASX) is a lipid-soluble xanthophyll carotenoid synthesized by many microorganisms and various types of marine life that is known to possess antioxidant, anti-inflammatory, antidiabetic, anti-atherosclerotic, and anticancer activities. As such, it is a promising nutraceutical resource. ASX-mediated modulation of PPARs and its therapeutic implications in various pathophysiological conditions are described in this review. ASX primarily enhances the action of PPARα and suppresses that of PPARβ/δ and PPARγ, but it has also been confirmed that ASX displays the opposite effects on PPARs, depending on the cell context. Anti-inflammatory effects of ASX are mediated by PPARγ activation, which induces the expression of pro-inflammatory cytokines in macrophages and gastric epithelial cells. The PPARγ-agonistic effect of ASX treatment results in the inhibition of cellular growth and apoptosis in tumor cells. Simultaneous and differential regulation of PPARα and PPARγ activity by ASX has demonstrated a hepatoprotective effect, maintaining hepatic lipid homeostasis and preventing related hepatic problems. Considering additional therapeutic benefits of ASX such as anti-gastric, cardioprotective, immuno-modulatory, neuroprotective, retinoprotective, and osteogenic effects, more studies on the association between ASX-mediated PPAR regulation and its therapeutic outcomes in various pathophysiological conditions are needed to further elucidate the role of ASX as a novel nutraceutical PPAR modulator.

## 1. Introduction

Peroxisome proliferator-activated receptors (PPARs) are members of the nuclear hormone receptors superfamily and are ligand-activated transcription factors [1]. They play an important role in the expression of many genes regulating cellular differentiation, the metabolism of glucose and lipids, and carcinogenesis [2,3,4]. Since the first PPAR was discovered in 1990 [5], three isoforms (PPARα, β/δ, and γ) have been identified in mammalian species [6]. PPARα is mainly expressed in the liver, kidney, heart, and skeletal muscle and is mainly involved in lipid metabolism and insulin sensitivity [7,8]. PPARβ/δ is ubiquitous throughout the human body and is responsible for epithelial cell growth, fatty acid oxidation, and wound healing, [9,10]. PPARγ is the most studied subtype and is found primarily in adipose tissues. In addition to its major role in glucose and lipid homeostasis, PPARγ is also associated with inflammatory responses, cardiovascular diseases, neurogenerative diseases, ocular diseases, and cancer [11]. Since these various physiological functions of PPARs can serve as therapeutic targets for the treatment of chronic diseases, researchers have studied synthetic and naturally occurring substances, as well as marine organisms, to identify specific ligands that modulate PPAR activities [12,13,14,15,16,17,18,19,20,21].

**Figure 1:**
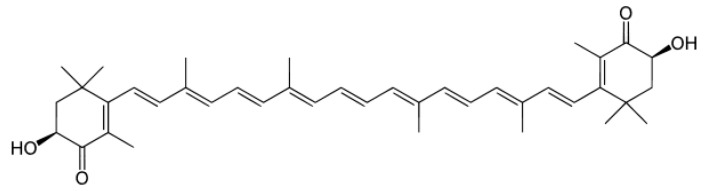
Chemical structure of astaxanthin (ASX).

**Figure 2:**
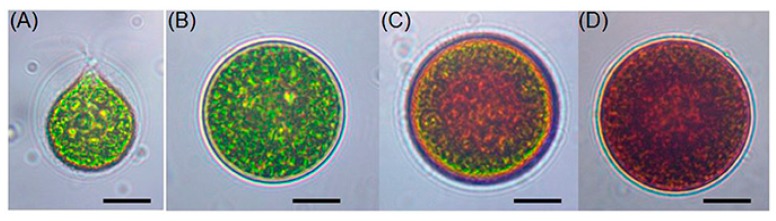
Microscopic appearance in *Haematococcus pluvialis* encystment. (**A**) green vegetative motile cell; (**B**) green vegetative palmella cell; (**C**) palmella cell accumulating ASX; (**D**) ASX fully-accumulated red aplanospore cell (from Shah et al. [26] distributed under the terms of the Creative Commons Attribution 4.0 International License (CC BY 4.0)).

Astaxanthin (ASX; 3,3′-dihydroxy-β,β-carotene-4,4′-dione) (Figure 1) is a lipid-soluble, red-orange-colored xanthophyll carotenoid synthesized by many microorganisms and various types of marine life [22]. ASX was first identified from *Astacus gammarus* (European lobster) in 1938 [23] and was initially approved as a food dye for salmon, trout, and shrimp feed in aquaculture industries [24]. Later in 1999, the United States Food and Drug Administration (USFDA) allowed the use of ASX as a dietary supplement [25]. A green microalga, *Haematococcus pluvialis*, is known to be a major producer of ASX. It accumulates up to 5% dry weight of ASX during encystment, which includes four progressive cellular morphological phases (Figure 2) [26]. Because synthetic ASX from petrochemicals exhibited inferior effects on human health when compared with natural algal-based ASX [27], many studies have been conducted on maximizing ASX production from *H. pluvialis* [28,29,30,31,32].

Numerous studies have demonstrated various biological properties and mechanisms of action of ASX. It displays antioxidant, anti-inflammatory, antidiabetic, anti-atherosclerotic, and anticancer activities and is a promising nutraceutical resource [33,34,35,36,37,38]. In this article, ASX-mediated PPAR modulation and its therapeutic implications are extensively reviewed and discussed.

## 2. Effects of ASX on PPAR Isoforms

### 2.1. PPARα

Most studies indicate that ASX acts as an agonist to PPARα [39,40,41]. Jia et al. [39] demonstrated that ASX significantly increased PPARα transactivation efficacy in PPARα-transfected Chinese hamster ovary (CHO-K1) cells; this effect was concentration-dependent. They also showed that ASX exhibits direct binding to human PPARα ligand binding domain with a K_D_ value (concentration at which a compound dissociates from the immobilized protein after the association phase) of 197 μM. The proportional sigmoidal increase in the time-resolved fluorescence resonance energy transfer (TR-FRET) ratio in agonist assay mode (the half-maximal effective concentration (EC_50_) = 3.9 μM) suggested that ASX plays a role as a ligand to activate PPARα. Furthermore, ASX significantly induced PPARα transcription and affected the expression of related target genes in HepG2 human hepatocellular carcinoma cells. In two other in vivo studies that used animals with a high-fat diet, administration of ASX alone [40] or in a combined treatment with flaxseed oil [41] showed a significant and dose-dependent increase in hepatic expression of the PPARα gene and protein. Meanwhile, a recent study [42] reported that ASX is predicted to suppress the expression of PPARα and its target molecules in the livers of mice with diet-induced nonalcoholic steatohepatitis (NASH).

### 2.2. PPARβ/δ

Because early studies have not identified any significant relationship between ASX and PPARβ/δ expression and/or activity [39,43], follow-up studies have not been conducted consistently. The relevant effect of ASX on PPARβ/δ was first confirmed by Kobori et al. [42] when they discovered that ASX significantly decreased mRNA expression of PPARβ/δ and related target genes in NASH mice. Another study by Rundblad et al. [44] also demonstrated that the intake of high-oleic sunflower oil (HOSO) with added ASX downregulates mRNA expression of genes associated with glucose and lipid metabolism, including PPARβ/δ, in the peripheral blood mononuclear cells (PBMCs) of healthy volunteers. Similar observations were observed in subjects receiving krill oil containing the same amount of ASX.

### 2.3. PPARγ

The physiological role of ASX in PPARγ expression and activity is quite complex. The association between ASX and PPARγ was first described in 2005 [45] when a specific PPARγ antagonist, GW9662, strongly inhibited ASX-induced expression of connexin 43, a protein related to early processes in carcinogenesis [46]. Similar observations were reported in ASX-treated K562 leukemia cells [47] and AGS cells (human gastric epithelial adenocarcinoma cell line) [48]. ASX significantly and dose-dependently induced cellular apoptosis and PPARγ protein expression in K562 cells, and these effects were attenuated by GW9662 [47]. Most recently, ASX was found to increase the expression and DNA-binding activity of PPARγ in *Helicobacter pylori*-infected AGS cells in a dose-dependent manner. In those same cells, ASX also improved catalase activity, inhibited intracellular and mitochondrial reactive oxygen species (ROS) levels, and diminished the gene expression of inflammatory cytokines that are suppressed by GW9662 co-treatment [48]. Another study by Kim et al. [49] also showed a significant increase in PPARγ mRNA levels along with other osteogenesis- and adipogenesis-related genes in ASX-treated neural stem cells.

In contrast, other studies have suggested that ASX may be a PPARγ antagonist. Jia et al. [39] reported that ASX dose-dependently inhibited PPARγ transactivation with a more than 16-fold higher K_D_ value (11.9 μM) compared with the value observed in PPARα. In the TR-FRET coactivator antagonist assay mode, the half-maximal inhibitory concentration (IC_50_) value for PPARγ was 607.8 μM. In addition, the expression of PPARγ and related genes was also significantly decreased by ASX treatment. Her et al. [50] used the ubiquitous transcription factor Yin Yang 1 (YY1)-transgenic zebrafish lines (GY), which are characterized by their induced expression of lipogenic genes, including PPARγ, associated with CCAAT-enhancer-binding protein (C/EBP) homologous protein 10 (CHOP-10) downregulation. The authors observed a marked decrease in PPARγ expression with preserved CHOP-10 levels in high-level GY-expressing (GY-H) larvae, which is characteristic of normal zebrafish lines. In an in vivo approach, high-dose ASX (30 μM) suppressed PPARγ expression and consequently influenced the mRNA levels of several genes involved in hepatic lipid metabolism in mice fed a high-fat diet [40].

Considering these conflicting outcomes, Inoue et al. [43] advanced a new theory that ASX acts as a selective PPARγ modulator (SPPARM) depending on the cell context. Among the various xanthophyll carotenoids, ASX only showed dose-proportional binding to PPARγ, with less binding affinity and lower maximum activation than the PPARγ full agonist rosiglitazone. Luciferase reporter gene assays using human embryonic kidney 293 (HEK293) cells as well as an evaluation of adipogenesis and target gene expression in 3T3-L1 adipocytes demonstrated that ASX has an antagonistic effect on PPARγ. Meanwhile, ASX treatment increased mRNA and/or protein expression of liver X receptor (LXR) and CD36 in a dose-dependent manner. Subsequently, ASX also increased the induction of ATP-binding cassette transporter ABCA1 and ABCG1 in thioglycollate-elicited peritoneal macrophages, which is attributable to the action of PPARγ agonists. The authors suggested possible benefits of ASX for the management of various chronic diseases, through the adaptive PPARγ-modulating effect.

## 3. PPAR-Related Therapeutic Implications of ASX

The biological and pathophysiological activities of ASX due to its regulation of PPARs are summarized in Table 1.

### 3.1. Anti-Inflammatory Effects

The anti-inflammatory properties of ASX have been described by many previous studies [33,37], and various molecular mechanisms of action have been suggested. These include a blockade of the nuclear factor kappa-light-chain-enhancer of activated B cells (NF-κB) signaling pathway [51,52,53,54,55,56], inhibition of c-Jun N-terminal kinase (JNK) in the mitogen-activated protein kinase (MAPK) signaling pathway [54,56], prevention of ROS accumulation by nuclear factor E2-related factor 2 (Nrf2) [55], positive modulation of Src homology region 2 domain-containing phosphatase-1 (SHP-1) protein expression [51], suppression of cyclooxygenase-2 (COX-2) and inducible nitric oxide synthase (iNOS) [57], and induction of heme oxygenase-1 (HO-1) [58].

Although more investigations are needed to clarify the association between PPARs and inflammation, it has been reported that PPARγ ligands can regulate inflammatory responses by the transrepression of several signaling pathways (NF-κB, activating protein 1 (AP-1), and signal tranducer and activator of transcription (STAT)-1) [59]. In agreement with this, ASX showed the induction of LXR and CD36 mRNA expression via PPARγ activation in macrophages [43]. In addition to their involvement in cholesterol and lipid metabolism, LXRs also suppress the expression of pro-inflammatory genes such as tumor necrosis factor α (TNF-α), COX-2, iNOS, and matrix metalloprotease 9 (MMP9) [60]. CD36, a class B transmembrane scavenger receptor, is expressed by multiple cell types including macrophages and plays an important role in the pro-inflammatory and oxidative pathways [61,62]. ASX also exhibited protective effects against *H. pylori*-induced gastric inflammation [48]. *H. pylori* induces the release and activation of inflammatory cytokines, such as interleukin (IL)-8, via NF-κB activation in gastric mucosa [63,64,65]. Subsequently, IL-8 stimulates the assembly of neutrophils and ROS generation in the infected lesion [66]. PPARγ activation by ASX treatment improved the activity of antioxidant enzyme catalase (a downstream target gene for PPARγ), restored ROS overproduction, and inhibited IL-8 expression in *H. pylori*-infected gastric epithelial cells.

Meanwhile, ASX-mediated reductions in plasma and hepatic TNF-α and IL-6 expression were reported in an in vivo study [40], which suggests the possible involvement of PPARα activation, but not of PPARγ activation.

### 3.2. Anticancer Effects

ASX has demonstrated anticancer activity through multiple mechanisms including cell growth inhibition, apoptosis induction, and interference of cell cycle progression [34]. Suggested molecular targets for ASX-induced cancer prevention and treatment include NF-κB, Janus kinase (JAK)/STAT-3, phosphatidylinositide 3-kinase/protein kinase B (PI3K/Akt), MAPK, Nrf2, and PPARγ [34]. Although some PPARγ agonists have shown pro-tumorigenic activities [67,68,69,70], PPARγ activation is also considered a promising therapeutic target for novel anticancer agents. This is based on its suppression of cellular growth and proliferation as well as its promotion of terminal differentiation and apoptosis [71,72,73].

As mentioned previously, a specific PPARγ antagonist (GW9662) suppressed ASX-mediated induction of the connexin 43 gene in mouse embryonic fibroblasts (C3H/10T1/2) [45]. This result implies that cancer-preventive upregulation of connexin 43 is associated with ASX and its ability to activate PPARγ. Zhang et al. [47] also demonstrated that ASX, in a time- and/or dose-dependent manner, inhibited cell growth, decreased cell viability, and induced cell cycle arrest and apoptosis in K562 leukemia cells, which are partly attenuated through the preincubation of GW9662.

In addition to PPARγ, the roles of other PPAR subtypes in carcinogenesis and chemoprevention have been demonstrated [4]. For example, PPARα-dependent hepatocarcinogenesis has been reported in chronic rodent models [74], and PPARα agonists have been found to attenuate cell growth and angiogenesis in various tumor strains including A459 human non-small cell lung cancer, B16-F10 murine melanoma, Lewis lung carcinoma, U87 human glioblastoma, and HT-1080 human fibrosarcoma [75,76]. There is limited proof that downregulation of PPARβ/δ by several antagonists can inhibit tumorigenesis; thus, there are still conflicting opinions on the relationship between PPARβ/δ and cancer development, treatment, and prevention [77,78]. Further comprehensive research on ASX-mediated PPARα or PPARβ/δ modulation and therapeutic impacts on cancer progression are necessary to elucidate its possible anticancer properties.

### 3.3. Effects on Lipid and Glucose Metabolism

All PPAR subtypes are involved in lipid and carbohydrate metabolism as well as the management of metabolic syndrome and related disorders such as obesity, type 2 diabetes, atherosclerosis, and non-alcoholic fatty liver disease (NAFLD) [11,79]. Most studies have demonstrated that the physiological role of ASX is mainly focused on hepatic lipid and glucose metabolism via the modulation of PPARα and/or PPARγ.

With its dual role as a PPARα agonist and PPARγ antagonist, Jia et al. [39] showed that ASX treatment decreases intracellular cholesterol and triglyceride contents in lipid-loaded HepG2 cells. Furthermore, ASX altered the expression of genes that target PPARα and PPARγ. It increased the expression of sterol carrier protein 2 (SCP2), acyl-CoA dehydrogenase very long chain (ACADVL), acyl-CoA dehydrogenase medium chain (ACADM), enoyl-CoA hydratase and 3-hydroxyacyl CoA dehydroganase (EHHADH), and sterol 27-hydroxylase (CYP27A1). It also decreased the expression of carnitine palmitoyltransferase (CPT) 2 and aconitase 1 (ACO1), which are involved in various lipid and glucose metabolism pathways. The results from hepatic transcriptome profile analyses were comparable to those of the commonly used hypolipidemic agents fenofibrate and lovastatin, suggesting a preventive role for ASX in metabolic disorders associated with hepatic hyperlipidemia like NAFLD or NASH. The in vivo effects of ASX on lipid metabolism were also assessed by the same laboratory [40], and they observed significantly increased PPARα and reduced PPARγ gene and protein expression in high-fat diet-fed mice with a co-treatment of ASX. Several PPARα and PPARγ-related target genes that play key roles in fatty acid uptake (caveolin-1), fatty acid β-oxidation (CPT1 and acyl-CoA oxidase (ACOX) 1), triglyceride hydrolysis (lipoprotein lipase (LPL)), and mitochondrial thermogenesis (uncoupling protein 2 (UCP2)) were also upregulated by ASX administration. The expression of lipogenic genes regulated by PPARα such as sterol regulatory element binding protein (SREBP) 1c, fatty acid synthase (FAS), and acetyl-CoA carboxylate 1 (ACC1) were not affected by ASX; however, LXRα, which may contribute to an increase in plasma high-density lipoprotein (HDL) cholesterol levels, was affected by ASX. In addition, ASX reduced hepatic steatosis through PPAR-mediated inhibition of the Akt-mTOR axis and activation of autophagy pathways. The authors concluded that ASX treatment ameliorates high-fat diet-induced hepatic lipid accumulation and hepatic steatosis in mice via differential regulatory actions involving PPARα and PPARγ. Another in vivo study by Xu et al. [41] investigated the effect of an ASX and flaxseed oil combination on hepatic lipid accumulation and oxidative stress. The combination resulted in the upregulation of PPARα protein expression and the increased mRNA expression of CPT1 and ACOX in high-fat diet-fed male rats. It also caused the downregulation of 3-hydroxy-3-methylglutaryl-CoA reductase (HMGCR) and SREBP1 proteins and decreased mRNA levels for FAS and ACC. These results could support the hepatoprotective properties of ASX and flaxseed oil confirmed by the reversal of hepatic steatosis, decrease in hepatic triglyceride and total cholesterol levels, and enhancement in liver antioxidant capacity.

In contrast, increased PPARγ expression was associated with the development of hepatic steatosis and lipotoxicity in GY zebrafish [50]. Overexpression of YY1 transcription factor caused yellow and greasy liver appearance as well as marked lipid accumulation in hepatocytes compared to control zebrafish lines. The mRNA expression levels were also increased in genes responsible for fatty acid synthesis, transport and binding, lipid storage, and hepatic lipogenesis. YY1-mediated suppression of CHOP-10 expression caused upregulation of C/EBPα and PPARγ and related target genes such as adipocyte protein 2 (aP2), caveolin-1, adiponectin, adipsin, and fat-specific gene 27 (FSP27). As a result of progressive hepatic steatosis, more than 90% of adult GY fish exhibited liver abnormalities (i.e., gross liver hypoplasia) and related lipotoxicity, which included increased lipid peroxidation, ROS generation, gene expression involving lipid β-oxidation, and lipo-apoptosis.

Kobori et al. [42] presented different results from those of previous studies and in which ASX was predicted to decrease the actions of PPARα and PPARβ/δ and reduce the mRNA levels of related genes in mice with diet-induced NASH. In addition, the expressions of PPAR target molecules such as patatin-like phospholipase domain containing 2 (PNPLA2) and promyelocytic leukemia protein (PML) were also affected by ASX. The authors suggested that the inhibitory effect of ASX on hepatic gene expression, which leads to reduced mitochondrial fatty acid oxidation, is attributable to the suppression of PPARα activity. Thus, further scientific studies are required to elucidate the molecular actions of ASX on PPAR function.

Several studies have also shown the effect of ASX on lipid and/or glucose control using other cellular resources. ASX completely inhibited rosiglitazone-induced lipid accumulation and reduced the mRNA expression of PPARγ target genes (aP2, fatty acid-binding protein, and LPL) in 3T3-L1 adipocyte [43]. In contrast, enhanced adipogenic differentiation and significant overexpression of PPARγ and other adipogenic genes were observed in ASX-treated neural stem cells [49]. More recently, Rundblad et al. [44] reported that the intake of ASX-added HOSO supplementation downregulates the expression of PPARβ/δ, as well as other genes affecting lipid and glucose metabolism, in PBMCs from healthy subjects.

## 4. Conclusions and Future Prospects

ASX exhibits significant anti-inflammatory and anticancer properties, and it helps regulate lipid and glucose metabolism based on its differential modulation of PPARs depending on the type of cells. Like other previously reported molecular mechanisms, ASX-mediated PPARγ activation induces the expression of many pro-inflammatory molecules in macrophages and gastric epithelial cells. Despite the conflicting views regarding the effect of PPARs on cancer, the agonistic effect on PPARγ by ASX treatment leads to the inhibition of growth and cell cycle as well as an induction of apoptosis against several tumor cells. The effects of ASX on lipid and glucose metabolism associated with PPAR modulation are somewhat complicated. Simultaneous PPARα activation and PPARγ suppression play a major role in hepatic lipid homeostasis as well as NAFLD and NASH, but the overexpression of PPARγ causes excessive hepatic lipid accumulation. Changes in PPAR expression also affect lipid and glucose metabolism in adipocytes and PBMCs.

PPARs are distributed ubiquitously and are involved in maintaining homeostasis and controlling diseases in human body. A variety of PPAR ligands have been discovered, and though they are known to play a role in metabolic disorders such as type 2 diabetes and dyslipidemia, research on their new therapeutic potential is ongoing because of their vast influence on human health. For example, PPARγ has been found to provide additional benefits for cardiovascular homeostasis and related functional problems including atherosclerosis, restenosis, and hypertension [80]. PPARγ also plays a beneficial role in neurodegenerative disorders such as Parkinsonism, amyotrophic lateral sclerosis, Alzheimer’s disease and brain injury, and ocular diseases [6,81,82]. Furthermore, PPARβ/δ has been implicated in cardiac function, epidermal biology, neuroprotection, and gastrointestinal tract functions [83,84,85,86]. Spurred by these findings, numerous clinical trials are underway, which are exploring PPAR-based therapies for the treatment of many diseases [87].

Considering that ASX has a wide range of biological activities not covered in detail in this review, including anti-gastric, cardioprotective, immuno-modulatory, neuroprotective, retinoprotective, and osteogenic effects [33,37], further comprehensive studies of ASX-mediated influence on PPAR activity and its therapeutic outcomes in various pathophysiological conditions are necessary to clarify the role of ASX as a novel nutraceutical PPAR modulator.

## Figures and Tables

**Table 1:** Summary of PPAR-related biological and pathophysiological activities of ASX.

Biological Activity	Effect on PPARs	Study Model	Study Result(s)	References
Anti-inflammatory	PPARγ↑	Thioglycollate-elicited peritoneal macrophages from C57BL/6J mice	Induced mRNA expressions of LXR and CD36	[43]
	PPARγ↑	*H. pylori*-infected AGS human gastric epithelial cells	Inhibition of *H. pylori*-induced increase in intracellular and mitochondrial ROS levels and IL-8 gene expression	[48]
	PPARα↑	High-fat diet-fed C57BL/6J male mice	Reduced mRNA expression and plasma and liver levels of TNF-α and IL-6	[40]
Anticancer	PPARγ↑	C3H/10T1/2 mouse embryonic fibroblast cells	Induction of connexin 43 expression	[45]
	PPARγ↑	K562 leukemia cells	Cellular growth inhibition, cell cycle arrest and induction of apoptosis	[47]
Lipid and glucose homeostasis	PPARα↑, PPARγ↓	Lipid-loaded HepG2 human hepatocellular carcinoma cells	Reduced cellular cholesterol and triglyceride contents; changes in target gene expressions for PPARα and PPARγ involved in lipid and glucose metabolism pathways	[39]
	PPARα↑, PPARγ↓	High-fat diet-fed C57BL/6J male mice	Altered expressions in several PPARα and PPARγ target genes; reduced hepatic steatosis	[40]
	PPARα↑	High-fat diet-fed Sprague-Dawley rats	Increased mRNA expressions in CPT1 and ACOX; decreased mRNA expressions in SREBP1, HMGCR, FAS, and ACC; reduced hepatic steatosis and hepatic triglyceride and total cholesterol levels	[41]
	PPARγ↑	YY1-transgenic zebrafish	Yellow and greasy appearance and marked lipid accumulation in the hepatocytes; increased mRNA expression of genes responsible for the fatty acid synthesis, transport and binding, lipid storage, and hepatic lipogenesis; upregulation of C/EBPα and PPARγ target genes; gross liver hypoplasia and related lipotoxicity	[50]
	PPARα↓, PPARβ/δ↓	C57BL/6J mice with high-cholesterol, high-cholate, and high-fat diet-induced NASH	Changes in PPAR target genes (inhibition of PNPLA2; activation of PML)	[42]
	PPARγ↓	3T3-L1 adipocytes	Inhibition of rosiglitazone-induced lipid accumulation; reduced aP2, FABP, and LPL mRNA levels	[43]
	PPARγ↑	Mouse neural stem cells	Increased lipid accumulation; overexpression of adipogenic genes	[49]
	PPARβ/δ↓	PBMCs from healthy volunteers	Downregulation of genes involved in lipid and glucose metabolism (including PPARβ/δ)	[44]

ACC, acetyl-CoA carboxylase; ACOX, acyl-CoA oxidase; aP2, adipocyte protein 2; ASX, astaxanthin; CPT1, carnitine palmitoyltransferase 1; FABP, fatty acid binding protein; FAS, fatty acid synthase; HMGCR, 3-hydroxy-3-methylglutaryl-CoA reductase; IL, interleukin; LPL, lipoprotein lipase; LXR, liver X receptor; NASH, nonalcoholic steatohepatitis; PBMC, peripheral blood mononuclear cell; PML, promyelocytic leukemia protein; PNPLA2, patatin-like phospholipase domain containing 2; PPAR, peroxisome proliferator-activated receptor; ROS, reactive oxygen species; SREBP1, sterol regulatory element binding protein 1; TNF-α, tumor necrosis factor α; YY1, Yin Yang 1 transcription factor.
